# Identification of Hypoxia Prognostic Signature in Glioblastoma Multiforme Based on Bulk and Single-Cell RNA-Seq

**DOI:** 10.3390/cancers16030633

**Published:** 2024-02-01

**Authors:** Yaman B. Ahmed, Obada E. Ababneh, Anas A. Al-Khalili, Abdullah Serhan, Zaid Hatamleh, Owais Ghammaz, Mohammad Alkhaldi, Safwan Alomari

**Affiliations:** 1 School of Medicine, Johns Hopkins University, Baltimore, MD 21287, USA; ybahmed180@med.just.edu.jo; 2 Faculty of Medicine, Jordan University of Science and Technology, Irbid 22110, Jordan; oeababneh185@med.just.edu.jo (O.E.A.); aaalkhalili181@med.just.edu.jo (A.A.A.-K.); aosarhan16@med.just.edu.jo (A.S.); zahatamleh181@med.just.edu.jo (Z.H.); oaghammaz189@med.just.edu.jo (O.G.); mralkhaldi160@med.just.edu.jo (M.A.); 3 Department of Neurosurgery, School of Medicine, Johns Hopkins University, Baltimore, MD 21287, USA

**Keywords:** glioblastoma multiforme, hypoxia, bioinformatics, IGFBP2, CP, LOX

## Abstract

**Simple Summary:**

This study developed a prognostic signature using hypoxia-related differentially expressed genes (DEGs) in Glioblastoma Multiforme (GBM) and identified three optimal gene signatures (CP, IGFBP2, and LOX) using multi-omics analysis. This was done using bulk and single-cell RNA sequencing to identify DEGs and integrated machine learning particularly LASSO regression to construct a prognostic model. Gene ontology and pathway analysis were used to study the biological processes affected by these genes. Additionally, gene enrichment analysis was incorporated to study the tumor microenvironment and drug sensitivity. An in-depth understanding of the complex biological pathways in GBM using this multi-omics approach is necessary to examine GBM’s behavior and prognosis presenting insights for potential therapeutic targets and survival outcomes of GBM patients.

**Abstract:**

Glioblastoma (GBM) represents a profoundly aggressive and heterogeneous brain neoplasm linked to a bleak prognosis. Hypoxia, a common feature in GBM, has been linked to tumor progression and therapy resistance. In this study, we aimed to identify hypoxia-related differentially expressed genes (DEGs) and construct a prognostic signature for GBM patients using multi-omics analysis. Patient cohorts were collected from publicly available databases, including the Gene Expression Omnibus (GEO), the Chinese Glioma Genome Atlas (CGGA), and The Cancer Genome Atlas—Glioblastoma Multiforme (TCGA-GBM), to facilitate a comprehensive analysis. Hypoxia-related genes (HRGs) were obtained from the Molecular Signatures Database (MSigDB). Differential expression analysis revealed 41 hypoxia-related DEGs in GBM patients. A consensus clustering approach, utilizing these DEGs’ expression patterns, identified four distinct clusters, with cluster 1 showing significantly better overall survival. Machine learning techniques, including univariate Cox regression and LASSO regression, delineated a prognostic signature comprising six genes (ANXA1, CALD1, CP, IGFBP2, IGFBP5, and LOX). Multivariate Cox regression analysis substantiated the prognostic significance of a set of three optimal signature genes (CP, IGFBP2, and LOX). Using the hypoxia-related prognostic signature, patients were classified into high- and low-risk categories. Survival analysis demonstrated that the high-risk group exhibited inferior overall survival rates in comparison to the low-risk group. The prognostic signature showed good predictive performance, as indicated by the area under the curve (AUC) values for one-, three-, and five-year overall survival. Furthermore, functional enrichment analysis of the DEGs identified biological processes and pathways associated with hypoxia, providing insights into the underlying mechanisms of GBM. Delving into the tumor immune microenvironment, our analysis revealed correlations relating the hypoxia-related prognostic signature to the infiltration of immune cells in GBM. Overall, our study highlights the potential of a hypoxia-related prognostic signature as a valuable resource for forecasting the survival outcome of GBM patients. The multi-omics approach integrating bulk sequencing, single-cell analysis, and immune microenvironment assessment enhances our understanding of the intricate biology characterizing GBM, thereby potentially informing the tailored design of therapeutic interventions.

## 1. Introduction

Glioblastoma multiforme (GBM) stands as the most common and lethal primary brain tumor among adults, comprising 14.5% of all central nervous system tumors and accounting for 48.6% of malignant central nervous system tumors. Despite notable progress in surgical techniques, radiotherapy, and chemotherapy, the prognosis of individuals with GBM continues to be poor, with a median survival time of approximately 14 months [1]. Hypoxia, a condition characterized by an insufficient supply of oxygen to tissues, represents a common attribute in solid tumors, including GBM. Within the context of GBM, hypoxia assumes a pivotal role in the advancement and progression of tumors by fostering tumor cell proliferation, stimulating angiogenesis, enhancing invasion potential, and conferring resistance to therapy. In addition, hypoxia has been shown to modulate the immune microenvironment in a way that results in the infiltration of immunosuppressive cells and the subsequent suppression of antitumor immune responses [2]. In the past decade, the rapid progression in high-throughput sequencing technologies has greatly revolutionized our ability to identify genetic alterations and gene expression changes linked to GBM. Many studies have reported the detection of genes that are differentially expressed in GBM, which potentially serve as valuable prognostic markers or therapeutic targets [3]. Nonetheless, the association between hypoxia and gene expression in GBM remains incompletely investigated. Therefore, in this study, we aimed to delineate the differentially expressed genes (DEGs) related to hypoxia within GBM patients using bulk and single-cell RNA sequencing data. Furthermore, our study was designed to delve deep into the intricate interplay and associations that exist between hypoxia-related genes and immune cell infiltration in GBM. Numerous machine learning models such as LASSO, Neural Networks, Naive Bayes, etc., have been used in studies aiding in pattern recognition and predictive analytics. Thus, we aimed to construct a machine learning model using LASSO for GBM diagnosis using hypoxia-related DEGs as prognostic factors for overall survival. We decided to utilize LASSO due to its capacity to handle high-dimensional data, reduce overfitting, and enhance interpretability. The results of this study may shed light on the underlying molecular mechanisms of GBM progression and unveil promising therapeutic targets for GBM.

## 2. Materials and Methods

### 2.1. Data Sources

**Figure 1:**
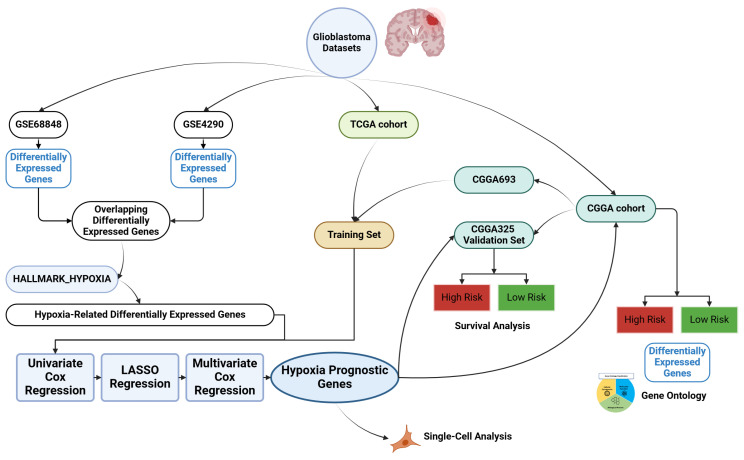
Flowchart of this study.

The flow chart in our study is presented in Figure 1. For the bulk sequencing analysis, patient cohorts were gathered from the Gene Expression Omnibus (GEO) (https://www.ncbi.nlm.nih.gov/geo/, accessed on 19 April 2023), the Chinese Glioma Genome Atlas (CGGA) (http://www.cgga.org.cn/, accessed on 19 April 2023), and The Cancer Genome Atlas—Glioblastoma Multiforme (TCGA-GBM) (https://portal.gdc.cancer.gov/projects/TCGA-GBM, accessed on 19 April 2023). Two GEO datasets (GSE68848, GSE4290) were identified. The GSE68848 dataset contained the expression data of 228 GBM patients and 28 non-tumor subjects, while GSE4290 included the expression data of 77 GBM patients and 23 non-tumor subjects. We manually collated the hypoxia-related genes (HRGs) from the Molecular Signatures Database (MSigDB) [4,5,6]. The expression data and clinical information for the TCGA-GBM cohort were acquired utilizing the R package “TCGAbiolinks” [7]. Furthermore, we manually extracted the expression data and clinical data of two CGGA datasets (CGGA325 and CGGA693) from the CGGA website. The CGGA693 data and the TCGA-GBM cohort were combined and used as the training set in our model. To remove batch effects, we applied the R package “sva” [8]. Following the exclusion of patients with a survival duration of less than 30 days, the combined dataset contained 388 GBM patients and the CGGA325 dataset, utilized as a validation set, included 135 GBM patients.

### 2.2. Identification of Hypoxia-Related Differentially Expressed Genes

For each bulk sequencing GEO dataset, we conducted a separate analysis to identify differentially expressed genes (DEGs) between GBM patients and non-tumor subjects. This analysis was performed using the GEO2R online analysis tool, which is an R-based web application integrated into the GEO database. The threshold of DEGs screening was $\left|\log_{2}\left(\mathrm{FC}\right)\right|\geq 1.5$ and p<0.05. Subsequently, we utilized the R package “EnhancedVolcano” to visualize the DEGs obtained from the two datasets through the generation of volcano plots. In addition, the common set of DEGs shared between the two datasets was isolated to intersect them with the gathered HRGs. The HRGs were extracted from the MSigDB manually and the intersect was visually represented utilizing the R package “VennDiagram” [9]. The resultant DEGs were then isolated for further analysis.

### 2.3. Construction of Machine Learning Diagnostic Model

Survival-associated genes that are concurrently linked to hypoxia were pinpointed through the application of univariate Cox regression (UCR). The Cox proportional hazards model explores the relationship of predictors and the time-to-event through the hazard function. The expression counts of the common sets of hypoxia-related DEGs were categorized into the high-expression tertile and the low-expression tertile for each patient, with middle-expression tertile patients being excluded from the analysis. These tertiles were subsequently used in creating a univariate Cox proportional hazard model using the R package “survival”. Survival-related DEGs were screened with a *p* < 0.01 as the criteria. This resulted in a list of survival-related DEGs that are also hypoxia-related. Then, the development of the least absolute shrinkage and selection operator (LASSO) model was carried out using the R package “glmnet” [10]. The expression counts of the survival-related DEGs that are also hypoxia-related were used as prognostic factors for overall survival (OS). LASSO regression eliminates unimportant variables via the regression coefficients penalizing the size of the parameters. LASSO regression shrinks the coefficient estimates toward zero, with the degree of shrinkage determined by an additional parameter, λ. To determine the optimal values for λ, a 10-fold cross-validation was utilized, thereby identifying prognostic genes and their coefficients. Cross-validation is a technique where the data are divided into multiple folds, with each fold being used to test the model and the rest for validation. This technique ensured that the model reliably predicts the outcomes and selects relevant variables even when applied to unseen data. The forest plot for the UCR and LASSO results was carried out with help from the R packages “ggplot2” and “patchwork”. Additionally, a multivariate Cox regression analysis was performed using the R package “survival”. The gene expression counts derived from the outcomes of the LASSO model were employed as the prognostic factors. The output genes and their coefficients were used in the development of hypoxia-related prognostic signatures.

### 2.4. Construction of Hypoxia-Related Prognostic Signature

The formula for hypoxia-related prognostic risk scores for each patient was:

$$\begin{array}{cc} \mathrm{Risk}\;\mathrm{score} & =\sum_{1}^{n}{\mathrm{coef}}_{i}\times x_{i} \end{array}$$

Here, “$x_{i}$” represents the expression level of each prognostic gene, and “coef*_i_*” signifies its respective coefficient. Based on the median value of the risk scores within each dataset, patients in both the training dataset and validation dataset were stratified into low-risk and high-risk groups. The heatmaps were generated to compare the expression between the two groups, employing the R package “pheatmap”. Kaplan–Meier survival curves were generated using the R packages “survival” and “survminer” to conduct a comparative analysis of overall survival (OS) between the high/low-risk groups. The R package “pROC” was employed for the display and analysis of ROC curves for the two groups [11]. In addition, we conducted both univariate and multivariate Cox regression analyses to assess the prognostic efficacy of the risk score model. These factors encompass gender, age, radiotherapy status, chemotherapy status, and presence of isocitrate dehydrogenase (IDH) mutation. The risk scores were obtained after combining both CGGA datasets and the TCGA-GBM data (batch effects were removed).

### 2.5. Gene Ontology (GO) and Kyoto Encyclopedia of Genes and Genomes (KEGG) Enrichment Analyses

The prognostic signature was applied to classify the two CGGA datasets (CGGA693 and CGGA325) patients into either high-risk patients or low-risk patients. Then, the R package “DESeq2” was employed to discern the DEGs between the high-risk group and the low-risk group. To gain insight into the biological functions and pathways affected by these DEGs, we employed R package “clusterProfiler” [12] to conduct GO and KEGG enrichment analyses on the DEGs (*p*-value < 0.05, q-value < 0.05). Data were retrieved using the R package “org.Hs.eg.db”. The outcomes of KEGG pathway analysis were visualized with the assistance of the R packages “GOplot” and “viridis”.

### 2.6. Tumor Immune Microenvironment and Drug Sensitivity Analysis

In both the CGGA and TCGA-GBM, the R package “estimate” was employed to compute the immune score and stromal score for each sample separately [13]. Next, we utilized the Transcriptome Integration and Multi-Omics Exploration Database (TIMEDB) platform [14] to assess the clinical significance of various immune cell populations in GBM. Clinical data and gene expression data were uploaded to the TIMEDB website for analysis. Figures and immune cells data were downloaded from the website. After that, *t*-test was employed to explore the association with risk.

Next, we explored the drug susceptibility in the two hypoxia-related genes risk groups and calculated the half-maximal inhibitory concentration (IC50) values of commonly used anticancer drugs using RNA-Seq of GBM-treated cell lines available through the Genomics of Drug Sensitivity in Cancer (GDSC) website using “oncoPredict” R package.

### 2.7. Single-Cell Sequencing Analysis

Single-cell RNA-seq analysis was carried out through the utilization of the Tumor Immune Single-cell Hub 2 web service, accessible at (http://tisch.compgenomics.org/home/, accessed on 19 April 2023). The analysis involved the utilization of the uniform manifold approximation and projection (UMAP) method to decrease dimensionality and visualize the clustering outcomes. Three glioma datasets were used in this study: GSE131928 10X, GSE131928 Smartseq2, and GSE141383. Additionally, to visualize the mRNA expression across distinct cell populations, UMAP distribution figures were generated. To understand the expression patterns of HRG at the anatomical level of GBM human tissues, we used the Ivy Glioblastoma Atlas, which is an anatomically based transcriptional atlas [15]. The anatomical locations were classified into: leading edge (tumor core), infiltrating tumor, cellular tumor, pseudopalisading cells, perinecrotic zone, hyperplastic blood vessels, and microvascular proliferation.

### 2.8. Statistical Analysis and Data Manipulation

In this study, all statistical analyses and data visualization were executed using the R statistical software 4.3.2. R provides a wide range of tools and packages for data analysis and visualization, which greatly contributed to the findings of this research. Specifically, packages such as “dplyr” and “ggplot2” were employed for data manipulation and advanced plotting, respectively. Leveraging this powerful software and these packages facilitated rigorous statistical analysis and enhanced the interpretability and visualization of the results.

## 3. Results

### 3.1. Identification of Hypoxia-Related Differentially Expressed Genes

**Figure 2:**
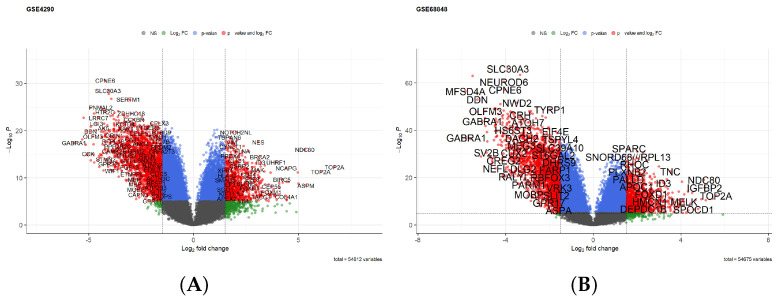

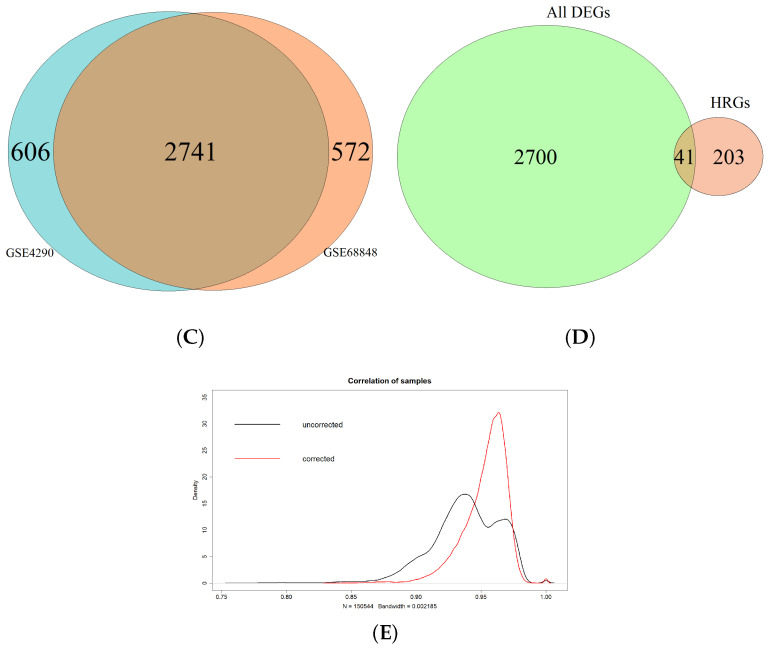
DEGs identification in different datasets of GBM. (**A**) Volcano diagrams of DEGs from the GBM vs. normal tissues in (**A**) GSE4290 dataset and (**B**) GSE68848 dataset. (**C**) Venn diagram showing the shared differential expressed genes in GSE4290 and GSE68848 datasets. (**D**) Venn diagram showing the 41 hypoxia-related genes (HRG) that are differentially expressed. (**E**) The pairwise correlation of the gene expression in the combined TCGA-GBM and CGGA693 (training set) before (black line) and after (red line) batch correction.

After identifying the DEGs of each GEO dataset using GEO2R, the resulting DEGs were screened with the threshold of an adjusted *p*-value < 0.05 and $\left|\log2\mathrm{FC}\right|>1.5$. After screening, 3313 DEGs (1318 upregulated and 1995 downregulated) were identified in GSE68848 and 3347 DEGs (1243 upregulated and 2104 downregulated) were identified in GSE4290. Volcano plots were employed for the visualization of the DEGs of the two datasets and are shown in Figure 2A,B. A total of 2741 overlapping DEGs were found between the two datasets as shown in Figure 2C. The 244 HRGs extracted from the MSigDB (Supplementary Table S1) were intersected with the 2741 DEGs to identify the hypoxia-related differentially expressed genes, of which 41 were found as depicted in Figure 2D. The list of these 41 hypoxia-related genes is shown in Supplementary Table S2.

### 3.2. Allocating the Training and Validation Datasets

The gene expression and clinical data of GBM patients from both the CGGA693 data and the TCGA-GBM cohort were merged. By applying batch effect removal procedures to the expression data, we decreased the inter-dataset variability. The pairwise correlation of the gene expression is higher as shown in Figure 2E. The resulting combined dataset was allocated to the training set, whereas the GBM patients of CGGA325 were allocated to the validation set. The training set contained 388 patients where 237 of them were males, and in which, the median age was 55 years. The validation set consisted of 135 patients where 85 were males with the median age of the whole set being 48 years.

### 3.3. Construction of Machine Learning Diagnostic Model

**Figure 3:**
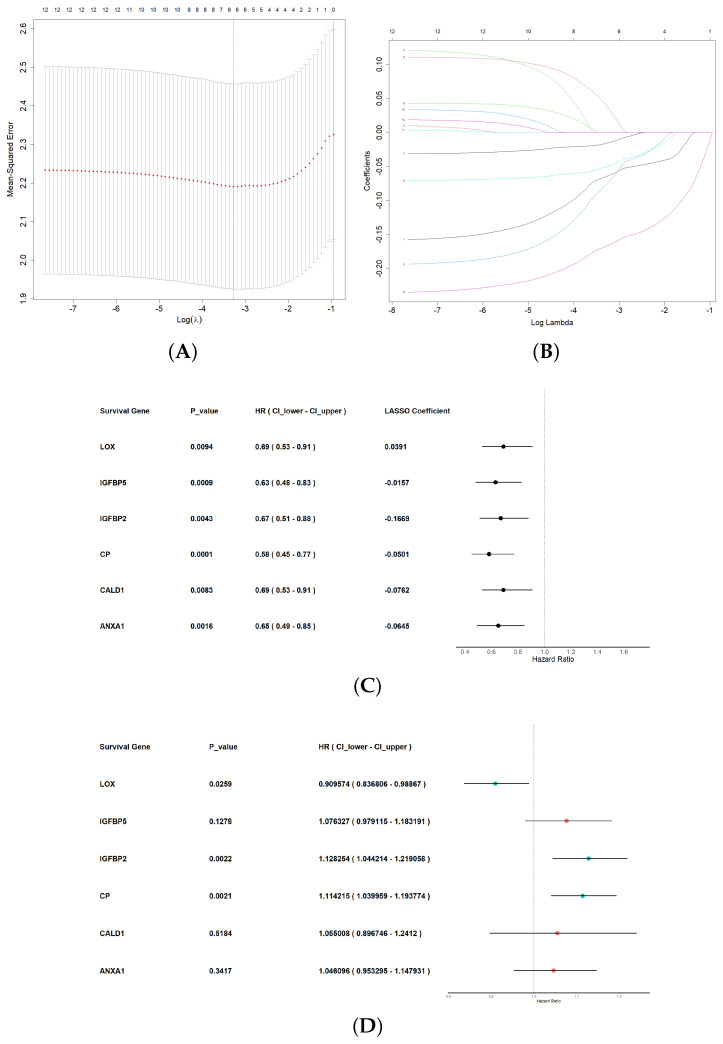
Construction of the prognostic risk score model based on a 3-hypoxia-gene signature in GBM. (**A**,**B**) Lambda and Lasso coefficients plots. (**C**,**D**) Univariate survival analysis of the 6 significant LASSO genes. (**D**) Multivariate Cox regression analysis of significant univariate genes.

To investigate the relation of the overall survival to the 41 hypoxia-related DEGs separately, we performed a univariate Cox regression analysis on the training dataset. This analysis considered the expression level (high or low) of these genes as the prognostic factor. We identified 12 DEGs that exhibited a significant relationship with the overall survival of GBM patients in the combined cohorts (*p* < 0.01). The 12 identified survival-related gene expression counts were used as prognostic factors in constructing the LASSO regression model. We generated a mean-squared error curve against the logarithm of the tuning hyperparameter λ. The dashed line represents the selected lambda value; a value of λ = 0.03768818 with log (λ) = −3.278409, Figure 3A. We selected the optimal λ value for the LASSO model through a 10-fold cross-validation. A coefficient profile plot (Figure 3B) was generated against the log (λ) sequence. Subsequently, training the optimized model using the training dataset resulted in six non-zero coefficients for “ANXA1”, “CALD1”, “CP”, “IGFBP2”, “IGFBP5”, and “LOX”. These genes and their coefficients are shown in Figure 3C alongside their univariate Cox regression results. Next, a multivariate Cox regression model was constructed incorporating these six candidate signature genes along with three optimal signature genes (“CP”, “IGFBP2”, and “LOX”)—with coefficients (0.1082, 0.1207, and −0.0948), respectively. The results are shown in Figure 3D.

### 3.4. Construction of Hypoxia-Related Prognostic Signature

**Figure 4:**
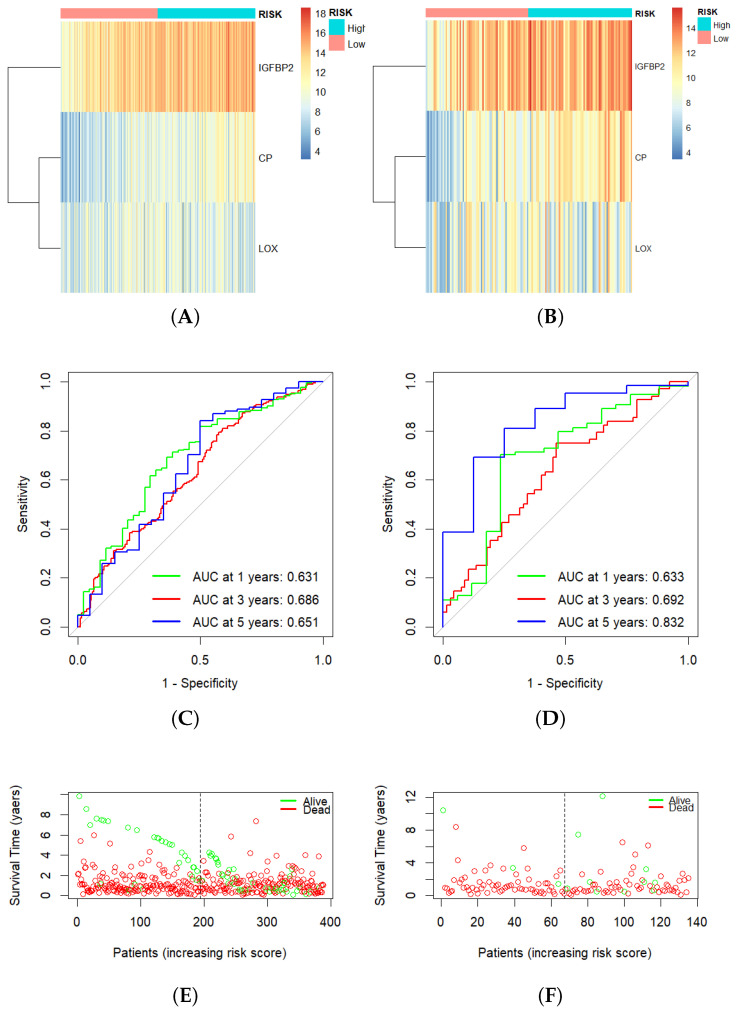
Construction of the HRG risk score model based on the significant 3-hypoxia-gene signature. Heatmap showing gene expression of IGFBP2, CP, and LOX in (**A**) training set and (**B**) validation set. (**C**,**D**) ROC curves based on 1-year, 3-year, and 5-year OS in training set and validation set. (**E**,**F**) Dot plot showing the relationship between the risk score value, survival time, and living status in training set and validation set, respectively.

**Figure 5:**
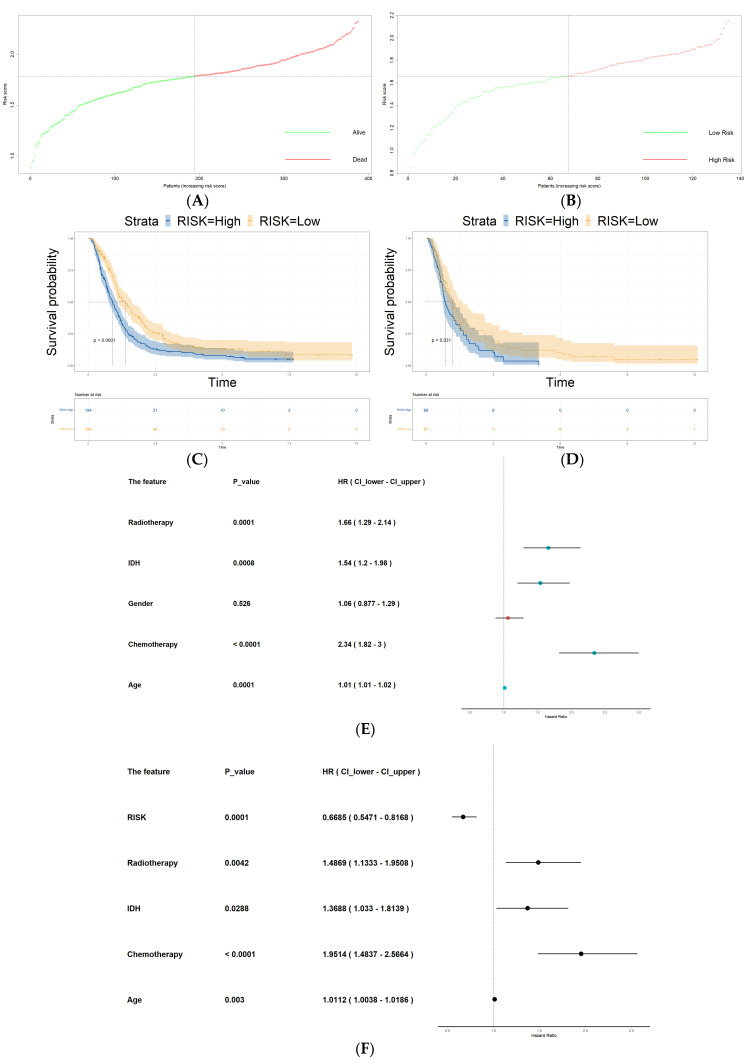
Survival validation of the HRG risk score model. (**A**,**B**) Dot plot showing the optimum cut-off of risk score based on median number of patients in training set and validation set, respectively. (**C**,**D**) Kaplan–Meier plots of OS showing significant worse survival in high-risk group in training set and validation set, respectively. (**E**) Univariate and (**F**) multivariate analysis of the combined datasets showing HRG risk score as an independent predictor of survival in GBM patients.

For each patient, the hypoxia-related prognostic risk scores were calculated using the following formula: Risk Score = 0.1082 × Expression (CP) + 0.1207 × Expression (IGFBP2) − 0.0948 × Expression (LOX). Utilizing the risk model, we computed the risk scores for both the training and validation sets. Subsequently, we categorized the samples into high- and low-risk groups employing the median risk score as the cut-off value. After that, we visualized the expression counts of the three signature genes in the two groups through heatmaps as displayed in Figure 4A,B. The risk scores for the training and validation sets are shown in Figure 5A, Figure 5B, respectively. The survival curves from both datasets indicated that the high-risk group had significantly shorter survival times compared to the low-risk group (*p* value < 0.001 for training and 0.033 for testing), Figure 5C,D. In the training set, the high-risk group exhibited a median overall survival (OS) of 0.81 years, whereas the low-risk group had a median OS of 1.16 years. The validation set high-risk patients showed an OS median of 0.81 years and the low-risk patients exhibited an OS median of 1.16 years. Furthermore, the ROC curve shows that, in the training set, AUC values for one-, three-, and five-year OS were 0.631, 0.686, and 0.651, respectively (Figure 4C). In the validation set, AUC values for one-, three-, and five-year OS were 0.633, 0.692, and 0.832, respectively (Figure 4D). The survival status in the training and validation sets are shown in Figure 4E, Figure 4F, respectively.

### 3.5. Risk Score as an Independent Prognostic Factor

Next, both the CGGA datasets and the TCGA-GBM dataset were combined, followed by the removal of batch effects. After obtaining the risk score for each patient within the combined dataset, we divided the patients into a high-risk group and a low-risk group based on their respective risk scores. Univariate Cox regression analysis on each of the clinicopathological features and risk groups showed that all of them have a role in survival except gender. The univariate Cox regression analysis results are shown in Figure 5E. After we identified the significant features from the univariate analysis, they were incorporated as prognostic factors in a multivariate Cox regression analysis. All features were found to be significant and are shown in Figure 5F.

### 3.6. Functional Annotations of DEGs between High-Risk and Low-Risk Groups

**Figure 6:**
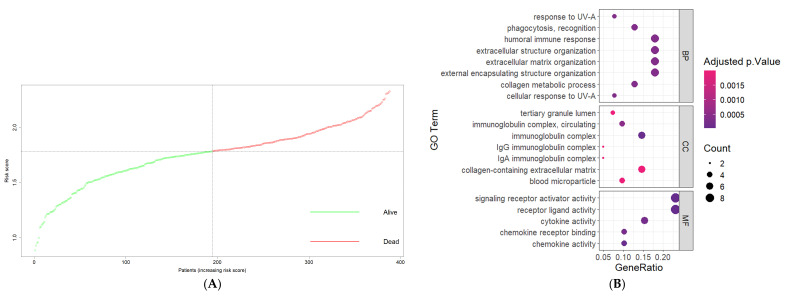

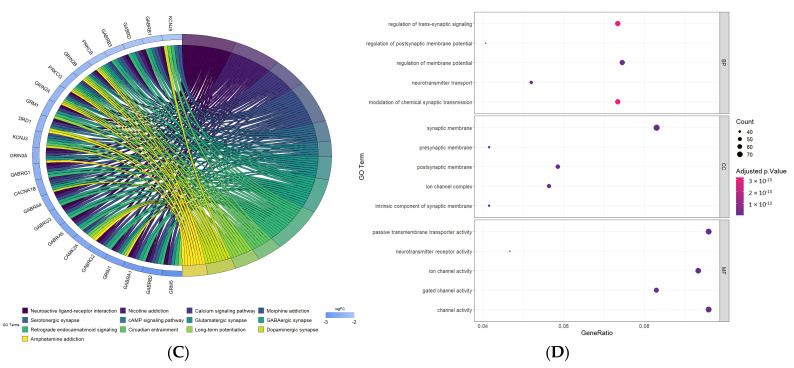
Bioinformatics analyses of DEGs between high-risk and low-risk patients. (**A**) Volcano plots of DEGs from high-risk vs. low-risk groups. (**B**) GO functions of the upregulated DEGs in high-risk patients. (**C**) GO functions of the downregulated DEGs in high-risk patients. (**D**) Circos plot showing the results of KEGG pathway enrichment analysis.

After the screening, we identified a total of 3807 DEGs in GBM patients from the CGGA cohort, with 54 of them upregulated and 3753 downregulated in the high-risk group compared to the low-risk group. These DEGs are shown in the volcano plot in Figure 6A. We further conducted gene ontology analysis separately for the upregulated genes and the downregulated genes. The top gene ontology terms for the upregulated genes regarding the biological processes were “cellular response to UV-A”, “phagocytosis, recognition”, and “collagen metabolic process”. These genes were notably enriched in cellular processes such as “immunoglobulin complex”, “immunoglobulin complex, circulating”, “IgG immunoglobulin complex”, and “IgA immunoglobulin complex”. As for the metabolic function, the genes exhibited enrichments in “receptor ligand activity”, “signaling receptor activator activity”, and “chemokine activity”. On the other hand, the downregulated genes were notably associated with biological processes like “neurotransmitter transport”, “regulation of membrane potential”, and “regulation of postsynaptic membrane potential”. Their predominant cellular components included “synaptic membrane”, postsynaptic membrane”, and “presynaptic membrane”. The notable metabolic functions for the genes were “gated channel activity”, “on channel activity”, and “neurotransmitter receptor activity”. The top terms of each aspect are visualized in Figure 6B,C. The KEGG pathway analysis revealed that the upregulated DEGs were closely associated with the “IL-17 signaling pathway”, the “Cytokine-cytokine receptor interaction” pathway, and the “TNF signaling pathway”. In contrast, the downregulated DEGs were found to be mostly associated with different signaling pathways, such as the “Neuroactive ligand-receptor interaction” pathway, the “Calcium signaling pathway” pathway, and the “cAMP signaling pathway”. Figure 6D shows the genes that are most associated with most of the pathways.

### 3.7. Tumor Immune Microenvironment and Drug Sensitivity Analysis

**Figure 7:**
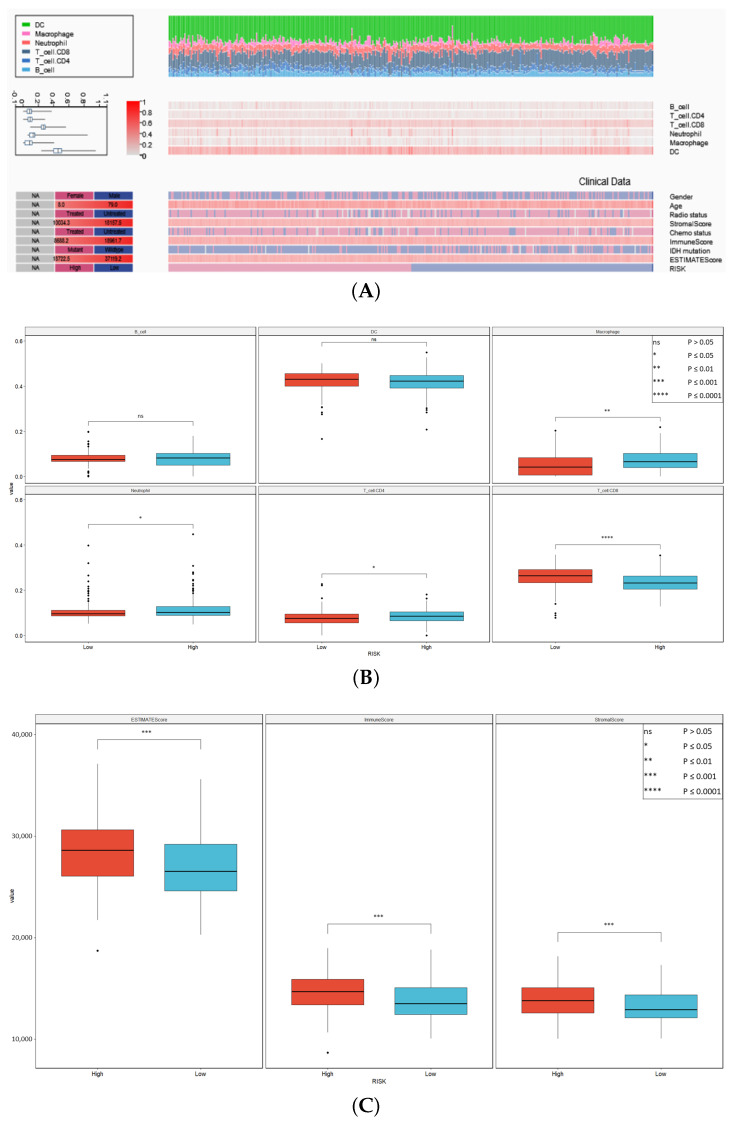
Tumor microenvironment analysis of CGGA patients. (**A**) Heatmap showing TME analysis and its relationship with other clinical and genomic variables. (**B**) Immune cell analysis based on TIMER algorithm between high- and low-risk patients. (**C**) Results of ESTIMATE algorithm showing significantly higher ESTIMATE, immune, and stromal scores in high-risk patients.

In the high-risk group of CGGA patients, stromal and immune scores turned out to be significantly higher. This trend was also seen in the impact of different immune cells (CD4 T-cell, CD8 T-cell, Neutrophil, Macrophage) shown in Figure 7A,B. The different immune cells’ infiltration in CGGA patients is seen in Figure 7C. As for the TCGA patients, no difference was noticed in the stromal score, immune score, or the impact of any of the immune cells.

The differences in drug susceptibility between the high- and low-risk groups were analyzed using a reference GBM-treated cell lines. There were 118 significantly different drugs between both clusters. Venetoclax, Cyclophosphamide, Dactinomycin, Lapatinib, and Palbociclib were the top five significantly different drugs with a lower IC50 values in the high-risk cluster, Figure S1A, while Talazoparib, Telomerase, GNE-317, Selumetinib, and AZD1332 were the genes with a lower IC50 in the low-risk cluster, Figure S1B.

### 3.8. Single-Cell Sequencing Analysis

**Figure 8:**
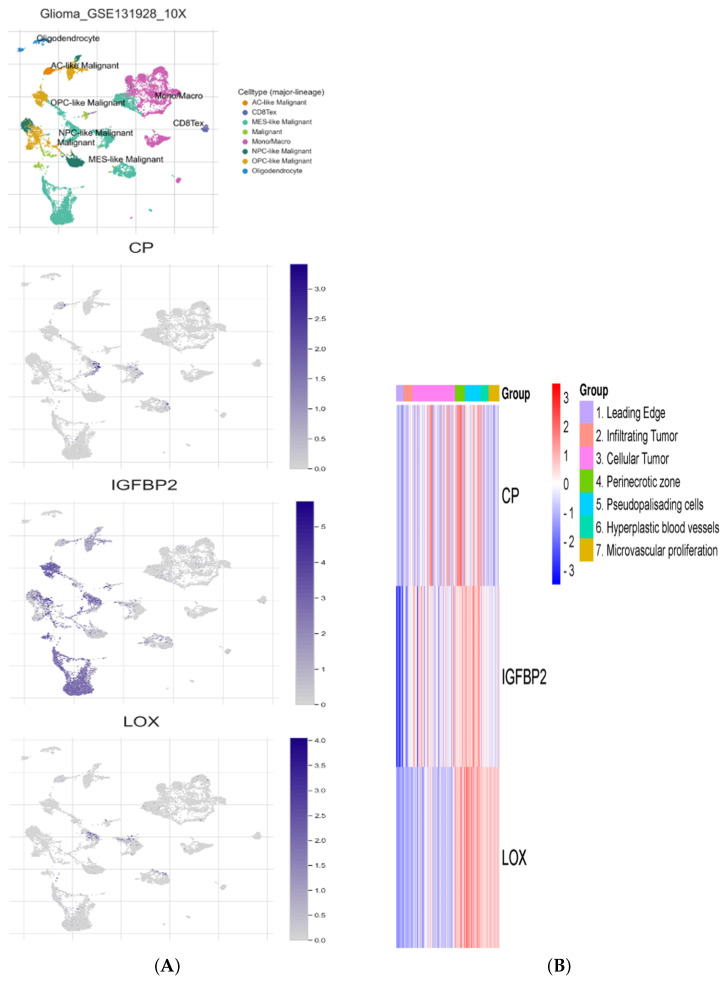
Results of single-cell RNA-seq analysis. (**A**) Single-cell analysis of GSE131928 showing singles of enrichment of CP, IGFBP2, and LOX genes in MES-like malignant cells. (**B**) Heatmap of the 3 HRGs according to the anatomical location of tumor sample using Ivy Glioblastoma Atlas showing that the expression of the 3 HRGs is highly related to the perinecrotic and pseudopalisading zones, while CP and IGFBP2 were also enriched toward the cellular zone and LOX was enriched toward the vascular zones.

Single-cell RNA-seq analysis of HRGs, including LOX, CP, and IGFBP2, was conducted across three distinct glioma datasets. Tumor-associated cells from these datasets were organized using the UMAP method to identify distinct expression patterns. In both the Glioma GSE131928 10X (Figure 8A) and Glioma GSE131928 Smartseq2 datasets, all genes demonstrated consistent expression in MES-like malignant cells. The gene IGFBP2 exhibited universal expression across all cell types, with the most pronounced levels observed in MES-like malignant and OPC-like malignant cells. Further examination of the Glioma GSE131928 Smartseq2 dataset revealed that IGFBP2 was also expressed in AC-like malignant and NPC-like malignant cells. In the context of immunotherapy, as observed in the Glioma GSE141383 dataset, all three genes were identified in malignant cells, Figure S2. However, IGFBP2 expression was also present in stromal and immune cells. We investigated the role of the three HRGs in the GBM tissue using the RNA-seq dataset provided by the Ivy Glioblastoma Atlas Project Figure 8B. All three genes were expressed in the perinecrotic and pseudopalisading zones. While IGFBP2 and CP displayed more expression in the cellular tumor zone toward the tumor center, LOX was expressed in the vascular regions at the periphery.

## 4. Discussion

Even with advancements in treatment, GBM patients continue to experience a median survival duration of merely 15 months. Hypoxia is considered one of the hallmarks of cancer and has been demonstrated to play a pivotal role in glioblastoma development, progression, and resistance to therapy. Understanding the interplay between the hypoxic tumor microenvironment and GBM behavior could provide valuable insights into potential therapeutic targets. This novel hypoxia prognostic signature in GBM integrated bulk sequencing, single-cell analysis, and tumor microenvironment assessment as a comprehensive approach that ensures a better understanding of the aggressive nature of GBM and hypoxia-mediated mechanisms in other tumors such as breast, lung, and colorectal [16,17,18]. The multi-omics approach contributes to the broader cancer field goals by offering new insights into GBM’s pathophysiology and therapeutic targets for drug development.

To this end, various studies have been conducted to establish a hypoxia gene signature with prognostic significance for GBM. In this study, we developed a prognostic signature consisting of three hypoxia-related genes to assess hypoxia characteristics linked to clinical prognosis in GBM. The selection of these genes stems from their differential expression in tumor tissues and their significant prognostic role in GBM.

There are multiple studies on the construction of a LASSO prognostic model for glioblastoma. Tong et al. [19] proposed a prognostic model of mitochondria and oxidative stress-related genes, whereas Zhang et al. [20] built a cuproptosis-related prognostic model, which can independently predict the prognosis of GBM patients. We also created a predictive model with a similar purpose. However, our model was constructed based on hypoxia-related genes.

Insulin-like growth factor binding protein 2 (IGFBP2) holds significant importance as a glioma oncogene acting as a central component in several oncogenic signaling pathways. It is considered as one of the most robust biomarker indicators of the aggressive nature of GBM [21]. IGFBP2 is involved in regulating signals that promote tumor progression, contributing to the pro-tumorigenic characteristics of cancer cells [22]. It also plays a role in regulating the activity of insulin-like growth factors (IGFs), which are important molecules involved in cell growth and differentiation. Previous studies suggested IGFBP2 as a key inducer of epithelial--mesenchymal transition (EMT) in many malignant cancers, making it a potential immunotherapy target in mesenchymal GBM [23,24,25]. A study conducted by Yuan et al. [26] established that elevated expression of IGFBP2 serves as an independent prognostic biomarker in GBM. It has been proven that cells with high expression of IGFBP2 tend to aggregate in the vicinity of focal necrotic regions within gliomas, which indicates an important role in hypoxia-related pathways [26,27]. Hypoxia-inducible factor 1α (HIF1α) primarily regulates genes involved in angiogenesis, metastasis, immune invasion, and radiation resistance [28]. Notably, IGFBP2 stimulates hypoxia-inducible factor 1α (HIF1α) expression. Interestingly, IGFBP2 and HIF1α have a reciprocal effect, where IGFBP2 stimulates HIF1α expression, and HIF1α promotes IGFBP2 expression under conditions of oxygen deprivation [29]. The reciprocal interaction between IGFBP2 and HIF1α is believed to exhibit a significant influence on the growth of GBM. These observations strongly suggest redirecting the focus toward targeting the HIF1α-IGFBP2 axis in the context of GBM.

Lysyl oxidase (LOX) is an enzyme that modulates the primary tumor microenvironment by stiffening the ECM and boosting the tumor’s ability to invade and metastasize [30,31,32]. LOX works by creating crosslinks between collagen and elastin within the ECM [33]. The active form of LOX is particularly effective at increasing the stiffness of the ECM [34]. This activation process depends on the involvement of BMP1, an enzyme responsible for converting LOX into the active form [35]. Donato et al. [36] reported a robust expression of LOX in serous ovarian cancer. Simultaneously, in vitro tests revealed that LOX might play a role in the initiation and progression of ovarian cancer by regulating cell proliferation, migration, and gene expression. In U87MG and A172 cell lines, the suppression of LOX through knockdown and its inhibition by BAPN in GBM cells had a notable impact on cellular migration, invasion, and the formation of soft agar colonies [37]. These findings substantiate the involvement of LOX in the process of the migration, invasion, and angiogenesis of astrocytoma. Survival analysis demonstrated that LOX and HIF1 were connected to astrocytoma prognosis, and functional investigations demonstrated that LOX might contribute to the initiation and progression of astrocytoma by regulating tumor cell proliferation and angiogenesis [37].

Ceruloplasmin (CP) is a versatile glycoprotein known for its involvement in various pathological conditions, both non-malignant and malignant. It plays crucial roles in diseases such as Wilson disease, inflammation, fibrosis, and neurodegenerative disorders [38,39]. CP has also been identified as a tumor promoter in various malignancies. Numerous studies have reported a positive association between serum CP levels and carcinogenesis, tumor stage, and recurrence in cancers like pancreatic cancer, oral cancer, lung cancer, leukemia, and Hodgkin’s lymphoma [40,41,42,43,44]. Moreover, high expression levels of CP are correlated with malignant potential in esophageal cancer, bile duct cancer, renal cell carcinoma, and adrenocortical carcinoma [45,46,47,48]. Expression of CP is significantly upregulated and associated with clinicopathological stage, disease occurrence, and poor outcomes in lung cancer patients [49]. CP influences oncogenesis-associated pathways, including the HIF-1 signaling pathway [50]. It is also responsive to variations in oxygen and iron concentrations and is upregulated during hypoxia [51,52,53]. Understanding the role of CP assumes particular relevance in the context of iron regulation and the radioresistant hypoxic environment. In a study conducted by Roy et al. [51], the radiation responses of two human GBM cell lines were analyzed. They found that CP showed significant downregulation at the transcript and protein levels. Interestingly, manipulating CP expression resulted in elevated levels of reactive oxygen species, increased superoxide anion levels, enhanced synthesis of SOD1, and alterations in cellular Fe2+ levels. This suggests that CP sensitizes GBM cells to radiation and that downregulation or low intracellular levels of CP may contribute to resistance against irradiation treatment.

The gene ontology analysis unveiled that the upregulated genes in high-risk patients were engaged in functions related to humeral adaptive immunity such as humoral immune response, IgA and IgG immune production, cytokine activity, and chemokine activity. In addition, these genes play a part in different RNA regulating systems such as RNA splicing and RNA interference systems. On the other hand, downregulated genes in high-risk patients were involved in neuronal processes such as synapse organization, neurotransmitter transport, and neurotransmitter receptor activity. Together these results suggest that high-risk patients have more immune modulatory activity and are more de-differentiated as reflected by the decreased neuronal functionality.

Gaining insight into the biology of glioblastoma might pave the way for earlier detection, a more favorable prognosis, and more accurate treatment prediction, all of which would improve the clinical outcome. The dismal prognosis of GBM is primarily correlated with intratumoral variability [54]. To address this issue, we conducted a single-cell RNA analysis of the three hypoxia-related genes. Neftel et al. [55] identified four molecularly distinct GBM subtypes, namely astrocyte-like (AC-like) cells, mesenchymal-like (MES-like) cells, oligodendrocyte progenitor-like (OPC-like) cells, and neural progenitor-like (NPC-like) cells. Interestingly, MES-like cells have been associated with high immune infiltration, NF1 mutation, and a hypoxic environment [55,56]. Our scRNA-Seq analysis revealed that all three hypoxia-related genes were enriched in the MES-like cells cluster. In addition, IGFBP2 was enriched in AC-like and NPC-like cells. Verma and Kondaiah found that IGFBP2 contributes to the stabilization and accumulation of cytoplasmic β-catenin [57]. Previous studies showed that β-catenin accumulation plays an important role in mesenchymal features and EMT [58,59,60]. LOX has also been identified as being upregulated in mesenchymal stem-like cells [61]. From a spatial point of view, we found that the three genes were expressed more in the perinecrotic and pseudopalisading zones. The perinecrotic zone refers to hyperproliferative areas that contain GBM stem cells [62]. Previous studies have shown that the hypoxic environment in the perinecrotic zone is very suitable for GBM stem cells’ survival and proliferation through the expression of HIF1 and HIF2 [63,64]. The pseudopalisading zone is typically seen in glioblastoma and describes an area of necrotic foci surrounded by hypercellularity; it is associated with aggressive tumor behavior [65]. Previous studies have shown that the pseudopalisading zone is also characterized as being a highly hypoxic region [66]. Brat et al. [67] hypothesized that pseudopalisades are hypoxic due to local vasoocclusive events by the angiopoietin-2-mediated activity of tumor cells, which leads to necrosis, hypoxia, and subsequent HIF1 upregulation. Interestingly, there were different expression patterns in the other areas, as CP as well as IGFBP2 were expressed more toward the cellular part of the tumor, whereas LOX was expressed more in the peripheral vascular zones. Such patterns may suggest different hypoxia-driving downstream mechanisms according to the spatial location in the tumor tissue.

It is important to be cautious when interpreting the findings of our study, given several limitations that need to be considered. First, the retrospective nature of the TCGA and CGGA datasets may introduce inherent biases and confounding factors. Prospective studies are necessary to corroborate and validate the prognostic value of our three genes scoring system in an independent cohort. Second, the functional roles and molecular mechanisms of these genes require additional exploration through in vitro and in vivo experiments. Third, the feasibility of translating the hypoxia gene signature into clinical practice warrants thorough evaluation, encompassing the availability of reliable assays and potential cost implications.

The hypoxia model presented in this study must be used as a complementary tool in patients with GBM as it offers a tool that can be used to stratify patients into different risk groups, aiming for more personalized treatment decisions. Clinically, patients presenting with high expression of these genes warrant more aggressive treatment strategies. However, this implementation warrants further validation in their ability to serve as therapeutic targets for immunotherapy development and their functional roles in GBM pathogenesis.

## 5. Conclusions

In summary, we built a three-hypoxia-gene model of IGFBP2, CP, and LOX, which are characterized as being prognostic and differentially expressed in GBM. These genes are mainly expressed in tumor cells based on our single-cell analysis. The results of our study establish a strong basis for subsequent comprehensive investigation of these genes in GBM.

## Data Availability

Data used in this study are publicly available from the gene expression omnibus (GEO) and cbioportal databases can be accessed through GSE91061 and GSE78220. The study workflow is shown in Figure 1.

## Supplementary Materials

The following supporting information can be downloaded at: https://www.mdpi.com/article/10.3390/cancers16030633/s1, Figure S1: Drug sensitivity analysis in high- and low-risk groups; Figure S2: Results of single-cell RNA-seq analysis in other datasets; Table S1: Genes related to hypoxia; Table S2: Hypoxia-related DEGs.

## References

[1] Grochans S., Cybulska A.M., Simińska D., Korbecki J., Kojder K., Chlubek D., Baranowska-Bosiacka I. (2022). Epidemiology of Glioblastoma Multiforme—Literature Review. Cancers.

[2] Park J.H., Lee H.K. (2022). Current Understanding of Hypoxia in Glioblastoma Multiforme and Its Response to Immunotherapy. Cancers.

[3] Wang A., Zhang G. (2017). Differential gene expression analysis in glioblastoma cells and normal human brain cells based on GEO database. Oncol. Lett..

[4] Subramanian A., Tamayo P., Mootha V.K., Mukherjee S., Ebert B.L., Gillette M.A., Paulovich A., Pomeroy S.L., Golub T.R., Lander E.S. (2005). Gene set enrichment analysis: A knowledge-based approach for interpreting genome-wide expression profiles. Proc. Natl. Acad. Sci. USA.

[5] Winter S.C., Buffa F.M., Silva P., Miller C., Valentine H.R., Turley H., Shah K.A., Cox G.J., Corbridge R.J., Homer J.J. (2007). Relation of a Hypoxia Metagene Derived from Head and Neck Cancer to Prognosis of Multiple Cancers. Cancer Res..

[6] Liberzon A., Birger C., Thorvaldsdóttir H., Ghandi M., Mesirov J.P., Tamayo P. (2015). The Molecular Signatures Database Hallmark Gene Set Collection. Cell Syst..

[7] Colaprico A., Silva T.C., Olsen C., Garofano L., Cava C., Garolini D., Sabedot T.S., Malta T.M., Pagnotta S.M., Castiglioni I. (2016). TCGAbiolinks: An R/Bioconductor package for integrative analysis of TCGA data. Nucleic Acids Res..

[8] Leek J.T., Johnson W.E., Parker H.S., Jaffe A.E., Storey J.D. (2012). The sva package for removing batch effects and other unwanted variation in high-throughput experiments. Bioinformatics.

[9] Chen H., Boutros P.C. (2011). VennDiagram: A package for the generation of highly-customizable Venn and Euler diagrams in R. BMC Bioinform..

[10] Friedman J.H., Hastie T., Tibshirani R. (2010). Regularization Paths for Generalized Linear Models via Coordinate Descent. J. Stat. Softw..

[11] Robin X., Turck N., Hainard A., Tiberti N., Lisacek F., Sanchez J.C., Müller M. (2011). pROC: An open-source package for R and S+ to analyze and compare ROC curves. BMC Bioinform..

[12] Wu T., Hu E., Xu S., Chen M., Guo P., Dai Z., Feng T., Zhou L., Tang W., Zhan L. (2021). clusterProfiler 4.0: A universal enrichment tool for interpreting omics data. Innovation.

[13] Yoshihara K., Shahmoradgoli M., Martínez E., Vegesna R., Kim H., Torres-Garcia W., Treviño V., Shen H., Laird P.W., Levine D.A. (2013). Inferring tumour purity and stromal and immune cell admixture from expression data. Nat. Commun..

[14] Wang X., Chen L., Liu W., Zhang Y., Liu D., Zhou C., Shi S., Dong J., Lai Z., Zhao B. (2023). TIMEDB: Tumor immune micro-environment cell composition database with automatic analysis and interactive visualization. Nucleic Acids Res..

[15] Puchalski R.B., Shah N., Miller J., Dalley R., Nomura S.R., Yoon J.G., Smith K.A., Lankerovich M., Bertagnolli D., Bickley K. (2018). An anatomic transcriptional atlas of human glioblastoma. Science.

[16] Chen A., Sceneay J., Gödde N., Kinwel T., Ham S., Thompson E.W., Humbert P.O., Möller A. (2018). Intermittent hypoxia induces a metastatic phenotype in breast cancer. Oncogene.

[17] Huang Z., Yang M., Li Y., Yang F., Feng Y. (2018). Exosomes Derived from Hypoxic Colorectal Cancer Cells Transfer Wnt4 to Normoxic Cells to Elicit a Prometastatic Phenotype. Int. J. Biol. Sci..

[18] Gu X., Zhang J., Shi Y., Shen H., Li Y., Chen Y., Liang L. (2021). ESM1/HIF-1*α* pathway modulates chronic intermittent hypoxia-induced non-small-cell lung cancer proliferation, stemness and epithelial-mesenchymal transition. Oncol. Rep..

[19] Tong S., Xia M., Xu Y., Sun Q., Ye L., Yuan F., Wang Y., Cai J., Ye Z., Tian D. (2023). Identification and validation of a novel prognostic signature based on mitochondria and oxidative stress related genes for glioblastoma. J. Transl. Med..

[20] Zhang B., Xie L., Liu J., Liu A., He M. (2023). Construction and validation of a cuproptosis-related prognostic model for glioblastoma. Front. Immunol..

[21] Phillips L.M., Zhou X., Cogdell D.E., Chua C.Y., Huisinga A., Hess K.R., Fuller G.N., Zhang W. (2016). Glioma progression is mediated by an addiction to aberrant IGFBP2 expression and can be blocked using anti-IGFBP2 strategies. J. Pathol..

[22] Yao X., Sun S., Zhou X., Guo W., Zhang L. (2016). IGF-binding protein 2 is a candidate target of therapeutic potential in cancer. Tumor Biol..

[23] Liu Y., Song C., Shen F., Zhang J., Song S.W. (2019). IGFBP2 promotes immunosuppression associated with its mesenchymal induction and Fc*γ*RIIB phosphorylation in glioblastoma. PLoS ONE.

[24] Yamini B. (2018). NF-*κ*B, Mesenchymal Differentiation and Glioblastoma. Cells.

[25] Cai J., Chen Q., Cui Y., Dong J., Chen M., Wu P., Jiang C. (2018). Immune heterogeneity and clinicopathologic characterization of IGFBP2 in 2447 glioma samples. OncoImmunology.

[26] Yuan Q., Cai H.Q., Zhong Y., Zhang M.J., Cheng Z.J., Hao J.J., Wang M.R., Wan J.H. (2019). Overexpression of IGFBP2 mRNA predicts poor survival in patients with glioblastoma. Biosci. Rep..

[27] Elmlinger M.W., Deininger M.H., Schuett B.S., Meyermann R., Duffner F., Grote E.H., Ranke M.B. (2001). In Vivo Expression of Insulin-Like Growth Factor-Binding Protein-2 in Human Gliomas Increases with the Tumor Grade. Endocrinology.

[28] Semenza G.L. (2011). Oxygen Sensing, Homeostasis, and Disease. N. Engl. J. Med..

[29] Lin K.W., Liao A., Qutub A.A. (2015). Simulation Predicts IGFBP2-HIF1*α* Interaction Drives Glioblastoma Growth. PLoS Comput. Biol..

[30] Cox T.R., Erler J.T. (2011). Remodeling and homeostasis of the extracellular matrix: Implications for fibrotic diseases and cancer. Dis. Model. Mech..

[31] Erler J.T., Weaver V.M. (2009). Three-dimensional context regulation of metastasis. Clin. Exp. Metastasis.

[32] Levental K.R., Yu H., Kass L., Lakins J.N., Egeblad M., Erler J.T., Fong S.F.T., Csiszar K., Giaccia A., Weninger W. (2009). Matrix crosslinking forces tumor progression by enhancing integrin signaling. Cell.

[33] Kagan H.M., Trackman P.C. (1991). Properties and function of lysyl oxidase. Am. J. Respir. Cell Mol. Biol..

[34] Ji F., Wang Y., Qiu L., Li S., Zhu J., Liang Z., Wan Y., Di W. (2013). Hypoxia inducible factor 1*α* -mediated LOX expression correlates with migration and invasion in epithelial ovarian cancer. Int. J. Oncol..

[35] Maruhashi T., Kii I., Saito M., Kudo A. (2010). Interaction between periostin and BMP-1 promotes proteolytic activation of lysyl oxidase. J. Biol. Chem..

[36] De Donato M., Petrillo M., Martinelli E., Filippetti F., Zannoni G.F., Scambia G., Gallo D. (2017). Uncovering the role of nuclear Lysyl oxidase (LOX) in advanced high grade serous ovarian cancer. Gynecol. Oncol..

[37] Silva R.d., Uno M., Marie S.K.N., Oba-Shinjo S.M. (2015). LOX Expression and Functional Analysis in Astrocytomas and Impact of IDH1 Mutation. PLoS ONE.

[38] Mukae Y., Ito H., Miyata Y., Araki K., Matsuda T., Aibara N., Nakamura Y., Matsuo T., Sakai H., Ohyama K. (2021). Ceruloplasmin Levels in Cancer Tissues and Urine Are Significant Biomarkers of Pathological Features and Outcome in Bladder Cancer. Anticancer Res..

[39] Scheinberg I.H., Gitlin D. (1952). Deficiency of ceruloplasmin in patients with hepatolenticular degeneration (Wilson’s disease). Science.

[40] Shah P.H., Venkatesh R., More C.B. (2017). Determination of role of ceruloplasmin in oral potentially malignant disorders and oral malignancy—A cross-sectional study. Oral Dis..

[41] Balmaña M., Sarrats A., Llop E., Barrabés S., Saldova R., Ferri M.J., Figueras J., Fort E., de Llorens R., Rudd P.M. (2015). Identification of potential pancreatic cancer serum markers: Increased sialyl-Lewis X on ceruloplasmin. Clin. Chim. Acta.

[42] Repetto O., Mussolin L., Elia C., Martina L., Bianchi M., Buffardi S., Sala A., Burnelli R., Mascarin M., De Re V. (2018). Proteomic Identification of Plasma Biomarkers in Children and Adolescents with Recurrent Hodgkin Lymphoma. J. Cancer.

[43] Li N., Hu H., Wu G., Sun B. (2019). Value of immune factors for monitoring risk of lung cancer in patients with interstitial lung disease. J. Int. Med Res..

[44] Zhevak T., Shelekhova T., Chesnokova N., Tsareva O., Chanturidze A., Litvitsky P., Andriutsa N., Samburova N., Budnik I. (2020). The relationship between oxidative stress and cytogenetic abnormalities in B-cell chronic lymphocytic leukemia. Exp. Mol. Pathol..

[45] Strickland N.J., Matsha T., Erasmus R.T., Zaahl M.G. (2012). Molecular analysis of Ceruloplasmin in a South African cohort presenting with oesophageal cancer. Int. J. Cancer.

[46] Han I.W., Jang J.Y., Kwon W., Park T., Kim Y., Lee K.B., Kim S.W. (2017). Ceruloplasmin as a prognostic marker in patients with bile duct cancer. Oncotarget.

[47] Zhu B., Zhi Q., Xie Q., Wu X., Gao Y., Chen X., Shi L. (2019). Reduced expression of ferroportin1 and ceruloplasmin predicts poor prognosis in adrenocortical carcinoma. J. Trace Elem. Med. Biol..

[48] Bleu M., Gaulis S., Lopes R., Sprouffske K., Apfel V., Holwerda S., Pregnolato M., Yildiz U., Cordo’ V., Dost A.F.M. (2019). PAX8 activates metabolic genes via enhancer elements in Renal Cell Carcinoma. Nat. Commun..

[49] Matsuoka R., Shiba-Ishii A., Nakano N., Togayachi A., Sakashita S., Sato Y., Minami Y., Noguchi M. (2018). Heterotopic production of ceruloplasmin by lung adenocarcinoma is significantly correlated with prognosis. Lung Cancer.

[50] Chen F., Han B., Meng Y., Han Y., Liu B., Zhang B., Chang Y., Cao P., Fan Y., Tan K. (2021). Ceruloplasmin correlates with immune infiltration and serves as a prognostic biomarker in breast cancer. Aging.

[51] Roy C., Avril S., Legendre C., Lelièvre B., Vellenriter H., Boni S., Cayon J., Guillet C., Guilloux Y., Chérel M. (2022). A role for ceruloplasmin in the control of human glioblastoma cell responses to radiation. BMC Cancer.

[52] Osaki S., Johnson D.A., Frieden E. (1966). The possible significance of the ferrous oxidase activity of ceruloplasmin in normal human serum. J. Biol. Chem..

[53] De Domenico I., Ward D.M., di Patti M.C.B., Jeong S.Y., David S., Musci G., Kaplan J. (2007). Ferroxidase activity is required for the stability of cell surface ferroportin in cells expressing GPI-ceruloplasmin. EMBO J..

[54] Shi T., Zhu J., Zhang X., Mao X. (2023). The Role of Hypoxia and Cancer Stem Cells in Development of Glioblastoma. Cancers.

[55] Neftel C., Laffy J., Filbin M.G., Hara T., Shore M.E., Rahme G.J., Richman A.R., Silverbush D., Shaw M.L., Hebert C.M. (2019). An Integrative Model of Cellular States, Plasticity, and Genetics for Glioblastoma. Cell.

[56] Kim Y., Varn F.S., Park S.H., Yoon B.W., Park H.R., Lee C., Verhaak R.G.W., Paek S.H. (2021). Perspective of mesenchymal transformation in glioblastoma. Acta Neuropathol. Commun..

[57] Verma B.K., Kondaiah P. (2020). Regulation of *β*-catenin by IGFBP2 and its cytoplasmic actions in glioma. J. Neuro-Oncol..

[58] Barzegar Behrooz A., Talaie Z., Jusheghani F., Łos M.J., Klonisch T., Ghavami S. (2022). Wnt and PI3K/Akt/mTOR Survival Pathways as Therapeutic Targets in Glioblastoma. Int. J. Mol. Sci..

[59] Coelho B.P., Fernandes C.F.d.L., Boccacino J.M., Souza M.C.d.S., Melo-Escobar M.I., Alves R.N., Prado M.B., Iglesia R.P., Cangiano G., Mazzaro G.L.R. (2020). Multifaceted WNT Signaling at the Crossroads between Epithelial-Mesenchymal Transition and Autophagy in Glioblastoma. Front. Oncol..

[60] Ang H.L., Mohan C.D., Shanmugam M.K., Leong H.C., Makvandi P., Rangappa K.S., Bishayee A., Kumar A.P., Sethi G. (2023). Mechanism of epithelial-mesenchymal transition in cancer and its regulation by natural compounds. Med. Res. Rev..

[61] Kim S.M., Lim E.J., Yoo K.C., Zhao Y., Kang J.H., Lim E.J., Shin I., Kang S.G., Lim H.W., Lee S.J. (2022). Glioblastoma-educated mesenchymal stem-like cells promote glioblastoma infiltration via extracellular matrix remodelling in the tumour microenvironment. Clin. Transl. Med..

[62] Schiffer D., Annovazzi L., Casalone C., Corona C., Mellai M. (2018). Glioblastoma: Microenvironment and Niche Concept. Cancers.

[63] Hardee M.E., Zagzag D. (2012). Mechanisms of glioma-associated neovascularization. Am. J. Pathol..

[64] Ishii A., Kimura T., Sadahiro H., Kawano H., Takubo K., Suzuki M., Ikeda E. (2016). Histological Characterization of the Tumorigenic “Peri-Necrotic Niche” Harboring Quiescent Stem-like Tumor Cells in Glioblastoma. PLoS ONE.

[65] Rong Y., Durden D.L., Van Meir E.G., Brat D.J. (2006). ‘Pseudopalisading’ Necrosis in Glioblastoma: A Familiar Morphologic Feature That Links Vascular Pathology, Hypoxia, and Angiogenesis. J. Neuropathol. Exp. Neurol..

[66] Giambra M., Di Cristofori A., Valtorta S., Manfrellotti R., Bigiogera V., Basso G., Moresco R.M., Giussani C., Bentivegna A. (2023). The peritumoral brain zone in glioblastoma: Where we are and where we are going. J. Neurosci. Res..

[67] Brat D.J., Castellano-Sanchez A.A., Hunter S.B., Pecot M., Cohen C., Hammond E.H., Devi S.N., Kaur B., Van Meir E.G. (2004). Pseudopalisades in Glioblastoma Are Hypoxic, Express Extracellular Matrix Proteases, and Are Formed by an Actively Migrating Cell Population. Cancer Res..

