# Supplementary material for: Identification of Hypoxia Prognostic Signature in Glioblastoma Multiforme Based on Bulk and Single-Cell RNA-Seq
Source: Cancers (Basel). 2024 Feb 1;16(3):633. doi: 10.3390/cancers16030633 (PMC10854729; doi:10.3390/cancers16030633)
Supplement: Supplementary file 1 [file cancers-16-00633-s001.zip › Supplementary (1).pdf]

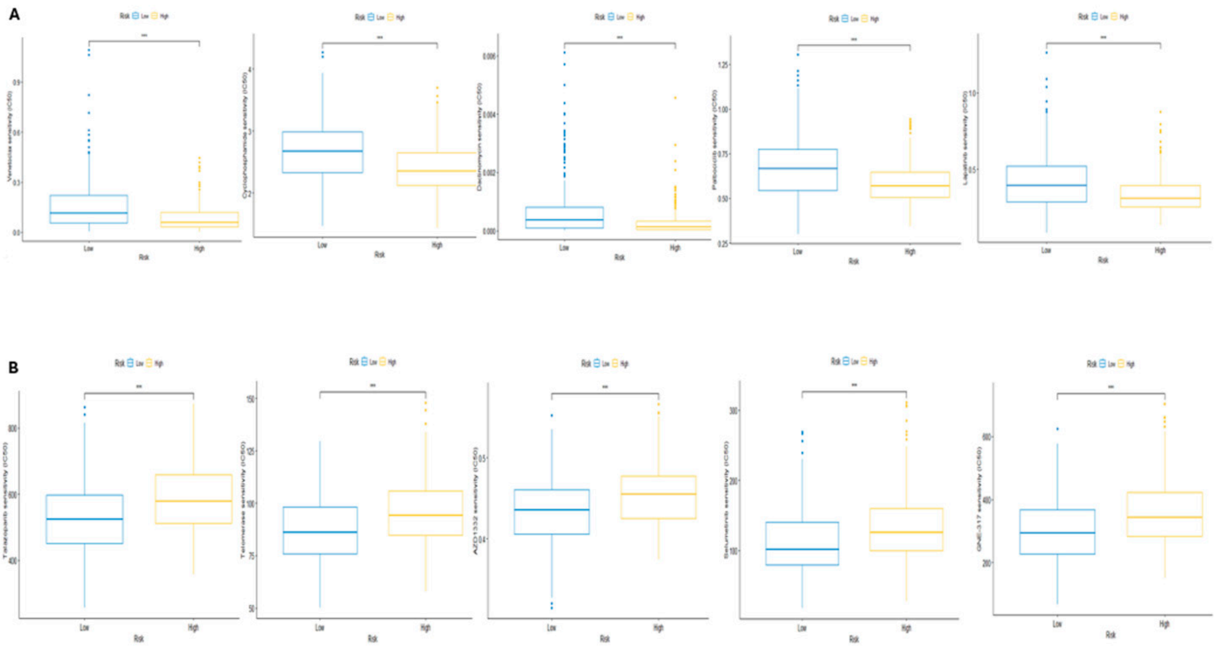

Figure S1. Drug sensitivity analysis in high- and low-risk groups. (A) Top 5 significantly different drugs with a lower IC50 values in the high-risk cluster. (B) top 5 significantly different drugs with a lower IC50 values in the low-risk cluster.

A

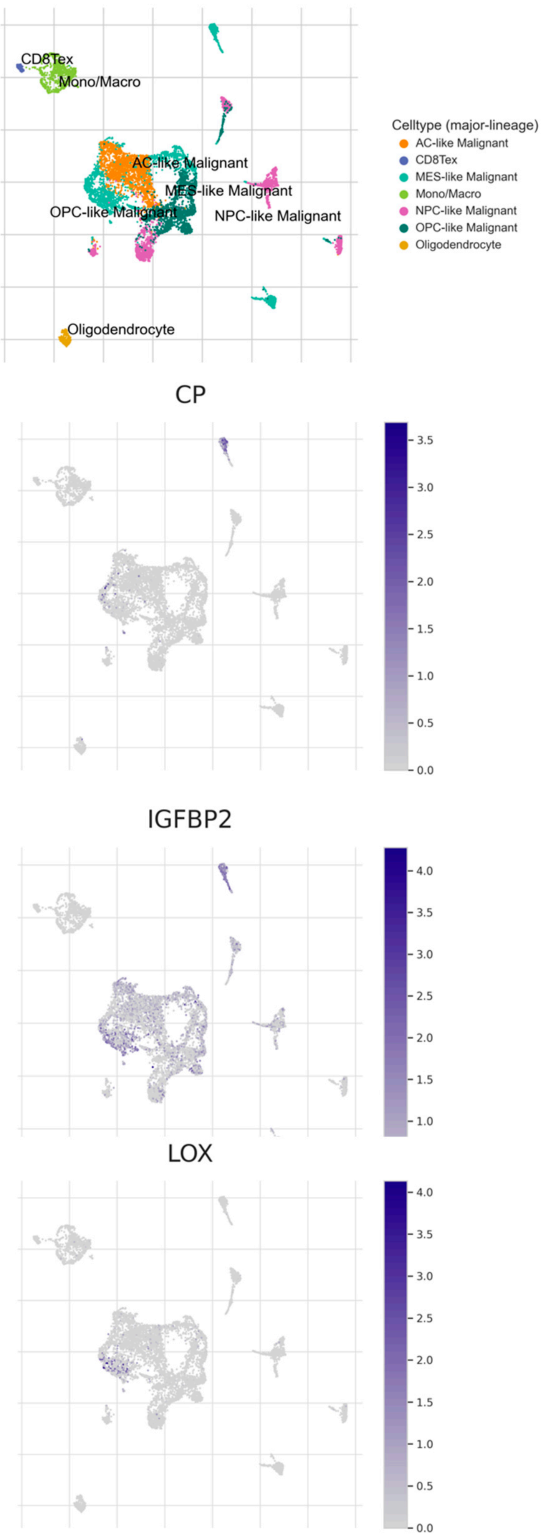
Glioma_GSE131928_Smartseq2
CD8Tex
Mono/Macro
AC-like Malignant
MES-like Malignant
OPC-like Malignant
NPC-like Malignant
Oligodendrocyte
Celltype (major-lineage)
AC-like Malignant
CD8Tex
MES-like Malignant
Mono/Macro
NPC-like Malignant
OPC-like Malignant
Oligodendrocyte
CP
IGFBP2
LOX

B

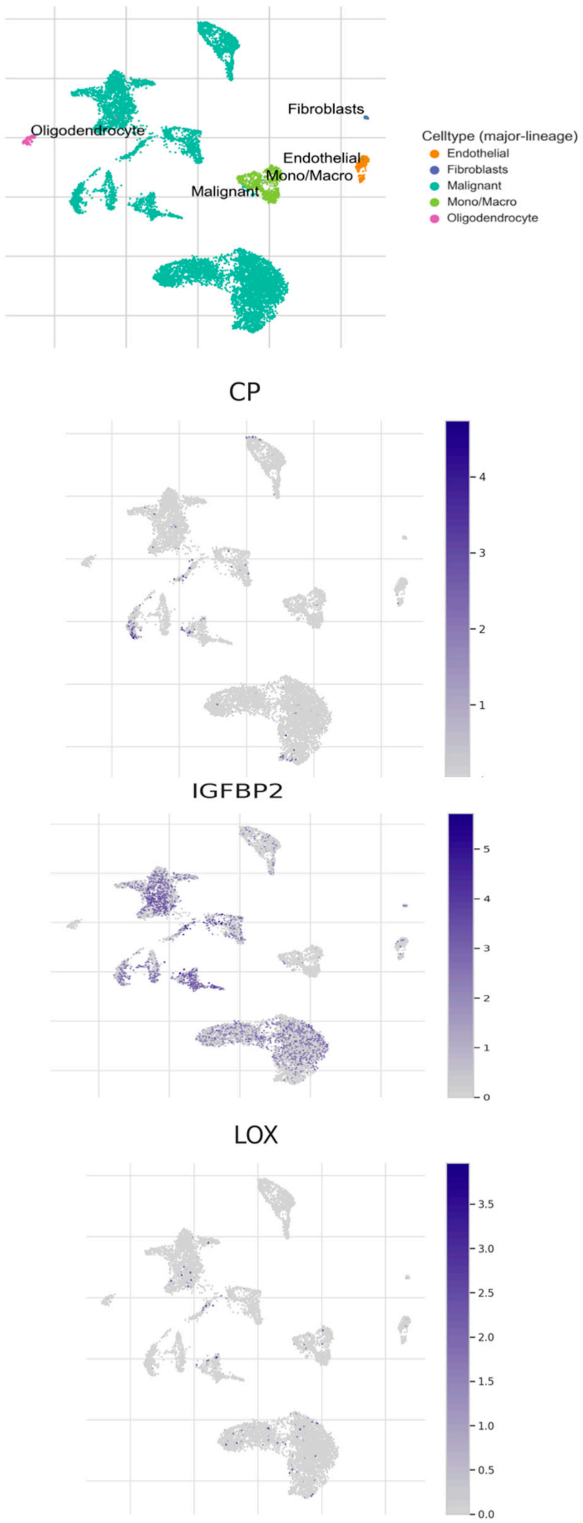
Glioma_GSE141383
Fibroblasts
Oligodendrocyte
Endothelial
Mono/Macro
Malignant
Celltype (major-lineage)
Endothelial
Fibroblasts
Malignant
Mono/Macro
Oligodendrocyte
CP
IGFBP2
LOX

Figure S2. Results of single-cell RNA-seq analysis in other datasets. (A) Single-cell analysis in GSE131928 showing singles of enrichment of CP, IGFBP2, and LOX genes in MES-like malignant cells. Singles for IGFBP2 and LOX genes in OPC-like and AC-like malignant cells are also present. (B) Single-cell analysis in the GSE141383 dataset which contains cells from GBM tissue in 6 patients treated with immunotherapy shows singles of enrichment of CP, IGFBP2, and LOX genes in malignant cells. There is a single of IGFBP2 from fibroblasts.
